# No Behavioral or ERP Evidence for a Developmental Lag in Visual Working Memory Capacity or Filtering in Adolescents and Adults with ADHD

**DOI:** 10.1371/journal.pone.0062673

**Published:** 2013-05-01

**Authors:** Marjolein Spronk, Edward K. Vogel, Lisa M. Jonkman

**Affiliations:** 1 Department of Cognitive Neuroscience, Maastricht University, Maastricht, The Netherlands; 2 Department of Psychology, University of Oregon, Eugene, Oregon, United States of America; University of Tokyo, Japan

## Abstract

Attention-deficit/hyperactivity disorder (ADHD) patients have both working memory (WM) and attention problems. Good attention skills are important for WM performance; individuals have higher WM capacity when being able to prevent storage of irrelevant information through efficient filtering. Since it is unknown how filtering ability is associated with WM performance in ADHD, this was investigated in the present study. A visuospatial working memory (VSWM) change detection task with distracting stimuli was administered to adolescents (12–16 years old) and adults (20–46 years old) with and without ADHD matched on education/IQ. Besides performance, contralateral delay activity (CDA) was measured; a neural correlate of the number of targets and distracters encoded and maintained in WM during the retention interval. Performance data showed similar WM-load, WM-distracter interference and developmental effects in ADHD and control groups. Adolescents’ performance on the WM task deteriorated more than that of adults in the presence of distracters and with higher WM-load, irrespective of Diagnosis. The CDA data suggested that initially all groups encoded/maintained distracting information, but only adults were able to bounce this information from memory later in the retention interval, leading to better WM performance. The only effect of Diagnosis was a smaller CDA in adolescents and adults with ADHD than in age/IQ-matched controls when maintaining a low 1-item load, which was possibly related to an inability to keep attention focused at cued stimuli with low task demands. Overall, the development of filtering efficiency and VSWM storage capacity in adolescents with ADHD was not different from that in typically developing peers.

## Introduction

Attention-deficit/hyperactivity disorder (ADHD) is a developmental disorder with a high prevalence of 6–9% in children [1], and is characterized by symptoms of inattention, impulsivity and hyperactivity (DSM-IV-TR) [2]. In approximately 80% of children these symptoms persist into adolescence [3]. Whereas hyperactivity and impulsivity symptoms tend to decline *most* with age, the cognitive problems related to inattention show the least reduction in adolescence [4]. An important cognitive deficit in ADHD is working memory (WM) impairment and several researchers have included WM as a significant cognitive factor in their theoretical models explaining the symptoms of ADHD [5], [6].

Impaired performance on neuropsychological memory tasks has been reported in children [6], [7], adolescents [8], [9] and adults [10]–[12] with ADHD and thus does not seem to resolve with age. Two meta-analysis studies reported deficits with large effect sizes in visuospatial short-term memory (VSSTM) or working memory (VSWM) in children, adolescents and adults with ADHD [13], [14]. Impairments in verbal STM and WM were also reported in ADHD but with smaller effect sizes than visuospatial deficits [13]. Whereas it is known that STM- and WM-capacity undergo development into adolescence [15] not many studies have investigated the development of WM performance in ADHD. However, WM-capacity is an important factor in the development of academic skills such as reading and mathematics [16] and fluid intelligence [17], [18] and therefore reduced WM-capacity in ADHD patients can have implications for academic achievement (e.g. in mathematics; [19]).

Some studies indicate that there might be a developmental delay especially in VSWM span in ADHD. In a cross-sectional study, it was found that VSWM impairments were present in younger (6–7 year-old) and older (8–12 year-old) children with ADHD combined-type (compared to typically developing children), whereas impairments in verbal WM were only found in the younger group [20]. Another study reported VSWM deficits in children with ADHD (compared to control children) that were largest during adolescence [9]. In these studies, ADHD and control groups were however not matched on IQ and since WM performance is highly correlated with IQ scores [21], [22], it is not clear to what extent these developmental effects were in fact caused by IQ differences.

An important question is to what extent a possible developmental delay in VSWM capacity in ADHD is related to inattention problems. Some studies have reported links between memory impairments and inattentive behavior in typical development as well as in ADHD. Other authors [23] showed that in 7–12 year-old children from a community sample, performance on WM tasks (verbal and visuospatial backward span) was related to inattentive behavior as reported by parents in a questionnaire. Another study [16] showed that in 6–16 year-old children higher inattention scores on the DSM-IV-TR [2] were associated with poorer visuospatial and verbal STM and WM. In ADHD, relations between memory and inattention symptoms have also been found. One study examined performance in phonological and visuospatial WM tasks in 8–12 year-old children with ADHD [24]. Attentive behaviour was measured by observation of orientation towards or away from the monitor or keyboard during respective presentation and response phases of the task. A larger increase in inattentive behaviour was found with increasing WM-load in children with ADHD than in typically developing controls. Results suggested that this was caused by a shortage of central executive resources in children with ADHD. Purely exceeding storage capacity decreased attentive behaviour to a similar extent in ADHD and control children.

The above studies all examined the relation between WM (or STM) performance and inattention symptoms in overt behavior (questionnaires, observation), but no studies so far have directly examined WM-attention relations in ADHD by manipulating both in one experimental task. This is important since the mechanisms by which attention influences memory performance or vice versa in ADHD are still unknown. Recent studies including healthy adult subjects have indicated that restrictions in VSWM-capacity can be related to filtering efficiency/selective attention deficits. In change detection tasks, VSWM-capacity is measured by presenting subjects with displays of varying numbers of items that have to be kept in memory for short periods of time, after which they are tested for memory accuracy [25], [26]. These tasks have revealed a maximum VSWM storage capacity of 3–4 items in adults [18], [27], [28]. Besides WM load, the relevancy of presented items was also manipulated in some studies by including distracters in the memory set (measuring filtering efficiency) [18], [27], [28]. These studies showed that an individual’s maximum VSWM-capacity was not only determined by the maximum number of items that can be kept in memory, but also by the ability to select relevant and ignore irrelevant items for maintenance in memory. If irrelevant information is stored in memory this will result in lower WM-capacity measures for tested items, since these items need space in WM. Hence, selective attention, e.g. the ability to enhance relevant information and suppress irrelevant information, plays a crucial role in determining which information enters WM and how much WM space is still available. Since impairments in selective attention or interference control are central to ADHD across all ages [29]–[34], they might be a significant factor in the lower VSWM-capacity often observed in ADHD patients.

In the present study a visuospatial change detection WM task with targets and distracters [28] was used to assess the development of VSWM-capacity and filtering efficiency in ADHD during adolescence. Besides performance measures, an electrophysiological measure called the contralateral delay activity (CDA; measured above the lateral parietal cortex) was obtained. The CDA is thought to be a direct neural measure of the number of items stored in WM since its amplitude has been found to increase linearly with the number of presented items and to reach a plateau when the maximum number of items a person can store in memory is exceeded [25], [26]. CDA amplitude does not only increase with the number of relevant items stored; similar amplitude rises can be found when irrelevant items (distracters) are stored in WM despite task instructions to ignore them. This was shown by Vogel et al. [28] who used a task in which subjects had to memorize the orientation of either two or four red items (the targets) for a later memory test. In a third condition two target items were accompanied by two distracter (e.g. blue-colored) items (T2D2; two targets, two distracters) that should not be stored in WM. The idea was that subjects with inefficient filtering abilities would also store the two distracter items in WM, having a total storage load of four items. Taken that the CDA amplitude varies with the number of stored items, this should be reflected by overlapping, or more similar, CDA’s in the T4D0 and T2D2 conditions than in T2D2 and T2D0. This was indeed what was found, but only in subjects with low WM-capacity. In subjects with high WM-capacity the T2D2-CDA overlapped with the T2D0-CDA and was smaller than the T4D0-CDA, showing that they had successfully prevented storage of the two distracter items. Thus, in this paradigm, the CDA can be used as a neural correlate of the number of relevant and irrelevant items that are encoded or maintained in memory in the retention interval of the task.

To our knowledge, this is the first time that the change detection task and the CDA-ERP measure are used to investigate the online storage of irrelevant and relevant information in VSWM in ADHD patients. Importantly, in the present study adolescents as well as adults with and without ADHD were included to study the developmental course of potential VSWM filtering and capacity deficits in ADHD. According to developmental lag theories of ADHD, cognitive deficits may be due to a developmental delay and persons with ADHD might eventually catch up with typically developing persons. For example, a developmental lag was previously proposed for inhibition [35], [36] and for verbal memory [20]. If a developmental lag for WM filtering and storage capacity indeed exists, larger differences could be expected between control adolescents and adolescents with ADHD than between adults with and without ADHD. Another important aspect of the present study is that ADHD and control groups were matched on IQ and educational level to exclude the possibility that group differences in WM-capacity or filtering efficiency are explained by IQ differences. The change detection task used in the present study closely resembles that used in previous change detection studies [26], [28], [37] and comprises three relevant stimulus conditions; a condition without distracters (T1D0) in which subjects store a low load of *one target* in WM, a *three target* condition without distracters (T3D0; storage of a higher load of three items) and a *one target* condition with *two distracters* (T1D2; storage of one item in case of perfect filtering). Based on earlier reported VSWM-capacity and attentional filtering deficits in ADHD, we expected larger unnecessary storage of distracter items in WM in ADHD patients, at least during adolescence. This would be reflected by 1) larger distracter interference effects on behavioral change detection measures (RT, accuracy, unnecessary storage) and 2) by larger T1D0-T1D2-CDA differences and/or smaller T1D2-T3D0 CDA differences in ADHD than control groups. The relation between unnecessary storage and WM-capacity will also be examined.

## Methods

### Participants

Originally 43 typically developing control subjects (23 adolescents and 20 adults) and 33 ADHD subjects (16 adolescents and 17 adults) participated in the study. Demographic characteristics of the control and ADHD adolescents and adults included in the study are presented in Table 1.

**Table 1:** Demographic characteristics of the control and ADHD adolescents and adults: number of participants (total, gender, ADHD-type) and means (standard deviations between brackets, range) of Age, YSR/ASR score, IQ and Forward, Backward and Standardized Digit span scores (WAIS III).

	N	male	female	Age	ADHD		YSR/ASR	IQ	Digit Span (verbal)		
					I	C			F	B	S
				14.8			53.1	96.5	8.3	5.3	8.7
Control	19	9	10	(1.4)	/	/	(4.3)	(7.3)	(1.7)*	(2.0)*	(2.9)*
Adolescents				Range:			50–67		Range:	Range:	Range:
				12–16					5–13	2–11	3–17
				14.8				99.1	7.5	5.3	8.3
ADHD	15	11	4	(1.0)	6	9	/	(11.6)	(1.6)*	(1.9)*	(2.8)*
Adolescents				Range:					Range:	Range:	Range:
				13–16					5–11	4–10	3–14
				31			52.7	97.9	10.0	8.1	12.1
Control	16	8	8	(8.8)	/	/	(3.9)	(9.3)	(2.3)*	(2.4)*	(3.4)*
Adults				Range:			50–63		Range:	Range:	Range:
				20–46					6–14	5–14	7–19
				28.2				103.00	8.8	7.2	10.2
ADHD	17	9	8	(5.9)	9	8	/	(10.0)	(1.9)*	(2.0)*	(2.9)*
Adults				Range:					Range:	Range:	Range:
				21–38					4–15	4–13	5–19

ADHD-I: inattentive type, ADHD-C: combined type.

YSR: Youth Self Report, ASR: Adults Self Report.

IQ; estimated IQ score from block design and vocabulary tests of the WISC and WAIS.

Digit Span: F = Forward, B = Backward, S = Standardized.

NB: stars indicate significant Age differences in Digit Span (*P* values): *P<.001.

#### Control subjects

The typically developing adolescents were recruited from a school providing secondary vocational education for 12- to 16-year-olds in order to match education backgrounds of control and to-be-recruited ADHD adolescents as much as possible. The adult control group consisted of a community sample recruited via advertisements in local newspapers, and were selected on having similar education backgrounds as the adolescents (no higher eduction). To confirm the absence of attention- and ADHD behavioral problems in the control subjects, all adolescents filled out the Youth Self Report (YSR) form [38] and all adults the Adult Self Report (ASR) form [39] (for mean scores see Table 1). Eight control participants (4 adolescents and 4 adults) were later excluded from the analysis due to several reasons. One control adolescent scored above threshold on the ADHD subscale of the Youth Self Report Form, a second adolescent could not perform the task (accuracy <55% in T1D0). One adult was excluded due to incomplete data, another due to scoring at chance level on % Hits in the easiest task condition T1D0 (i.e. 59%). The additional four control subjects (2 adolescents and 2 adults) were excluded due to IQ scores not falling within an IQ inclusion range of 80–120. This upper range of 120 was necessary to be able to match ADHD and non-ADHD groups on IQ. The remaining groups consisted of 19 control adolescents and 16 control adults. The control subjects were recruited and measured earlier than the ADHD subjects and performance and CDA data from these (albeit somewhat larger) control groups are presented in a developmental paper [40] about typical development of filtering in WM (CDA data only being partly overlapping with those presented in this paper).

#### ADHD subjects

All ADHD participants were recruited via the Regional Institute for Ambulant Mental Health Care (RIAGG) in Maastricht (the Netherlands) and had an official DSM-IV-TR diagnosis [2] of combined or inattentive ADHD established by a psychiatrist for at least 6 months prior to the start of the study (for numbers of inattentive and combined diagnoses see Table 1). To ensure the presence of childhood inattention problems in adults with ADHD, in addition to the DSM-IV-TR-diagnosis, retrospective reports of the presence of ADHD symptoms in childhood were assessed by a Dutch DSM-IV based self-report ADHD questionnaire [41]. This questionnaire was also filled in for the current situation. A positive response on respectively 7 and 8 out of 9 questions of the inattention subscale confirmed the presence of inattention problems in childhood (before the age of 7) and at the moment of testing. Due to an administrative error retrospective scores from two adults were missing. All subjects in the ADHD groups were free from other DSM-IV comorbidity, except for three adolescents with ADHD that had a comorbid diagnosis of Oppositional Defiant Disorder (ODD). Of the 16 adolescents, one was later excluded from the analysis because of chance performance already in the easiest task condition (accuracy <55% in T1D0). The remaining ADHD groups consisted of 15 adolescents and 17 adults. All ADHD participants on medication (only Ritalin or Concerta was used in this group) refrained from taking their medication for a period of 24 hours before the start of the experimental session. To derive an estimated IQ score, all adolescents were administered two subtests of the Wechsler Intelligence Scale for Children (WISC-III) [42]; Vocabulary and the Block Design tests. Adults performed the same subtests from the Wechsler Adults Intelligence Scale (WAIS-III) [43]. The estimated IQ score based on these two subtests has a mean reliability and validity of.9 [44], [45]. IQ-scores did not differ significantly between groups (F(1,63) = 1.5, *P* = .230).

The present study was approved by the Medical Ethics Committee of the azM and Maastricht University (MEC azM/UM) in the Netherlands, and prior to the study a written informed consent was obtained from the children and their caretakers and from the adults according to the Declaration of Helsinki (1964). All subjects were paid for their participation in the experiment.

### Procedure

The experimental session lasted 2.5–3 hours. The session started with three tests from the WISC-III for adolescents and WAIS-III for adults; the block design test, vocabulary test and the digit span test. The latter test (digit span forward and backward) was performed by the participants to obtain an independent standardized measure of verbal short-term and WM-capacity. Subsequently, the electrodes were attached. During the experimental session all participants sat in front of a 17-inch VGA monitor with their eyes aligned to the centre of the screen at a distance of approximately 75 cm. The participants were instructed to minimize eye blinks and to refrain from making head or eye movements during task performance. The experimental session started when all tasks were practiced until a predetermined performance criterion (75% correct responses) was reached.

### Experimental Task and Stimuli

**Figure 1:**
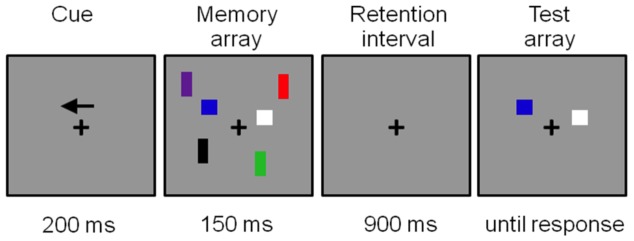
Example of distracters-present trial (T1D2) for the left hemifield.

To measure differences in WM-capacity and the efficiency of excluding irrelevant items from memory between Age and Diagnosis groups, a VSWM task comparable to that used in earlier change detection studies [26], [28] was presented to the subjects. This task consisted of bilateral stimulus displays in which colored squares (0.76°×0.76°) or rectangles (1.15°×0.57°) were presented within two 4°×7.3° rectangular regions presented 3° to the left and right from of a central fixation cross; see Fig. 1). On each trial, the positions of the items were randomly distributed within upper and lower quadrants of the screen with the constraint that the distance between objects within a hemifield was at least 2° (centre to centre). The colour of squares and rectangles was randomly selected on each trial with limited replacement from a set of seven easily distinguished colours (red, blue, green, violet, yellow, black and white). The number of targets and distracters was always the same in both hemifields, only location and color of the stimuli could differ between hemifields. All stimuli were presented on a gray background.

A trial started with the presentation of an arrow cue that indicated the hemifield that subjects should attend to on the following memory display. The subject’s task was to remember the locations and colors of the squares (targets: T) in this cued hemifield for a later test. A total number of 480 trials was presented. On half of the trials the squares were accompanied by distracters (D: colored rectangles) that had to be ignored. In total, there were four different types of memory displays differing in the number of targets and distracters. Either one or three targets (squares) were presented alone (T1D0 or T3D0; memory load of 1 or 3 items) or were accompanied by two distracters (T1D2 or T3D2). The T3D2 condition exceeded maximum capacity and was included to obtain equal numbers of trials with or without distracters. All memory displays were followed by a test display 900 ms later in which one colored square was presented at one of the locations in the preceding memory display within the upper or lower quadrant (to both hemifields). The subjects had to press a left button with the left index finger when the test stimulus shown at this location had the same color as that in the previous memory display (50% of all trials) or press right with the right index finger when it was different. A new memory display followed 500–700 ms after a response was given. See Fig. 1 for an example of a complete trial of the T1D2 condition with exact timing parameters.

The behavioral measures derived from the VSWM change detection task in T1D0, T1D2 and T3D0 were: 1) reaction times for correct detections (RT), 2) percentage correct responses (% Hits), and 3) *K*-scores (see below). Only reaction times to correct responses that fell within a response window from 250–4000 ms after the memory probe were included in the analysis. Cowan’s memory capacity measure *K* in T1D0 and T3D0 conditions was computed with a standard formula (Cowan, 2001): *K* = (H+CR –1) N, in which H is the hit rate, CR are the correct rejections in an array with N items. To derive a behavioral measure of filtering efficiency, following a study by (Lee et al., 2010) we also computed *K* in the distracter condition (T1D2) by filling in 1 for N since there was 1 target item; if distracters are perfectly filtered out *K* will be 1, in case of imperfect filtering *K* will be lower than 1. This *K*-T1D2 measure was subtracted from *K*-T1D0 to obtain an “unnecessary storage” measure.

### Electrophysiological Recording and Analysis

For measurement of the EEG, an elastic cap (Easycap) containing 60 Ag/AgCl electrodes was used. The montage included 7 midline sites (Fpz, Fz, FCz, Cz, CPz, Pz, Oz), and 52 lateralized sites (Fp1, Fp2, AF7, AF3, AF4, AF8, F7, F5, F3, F1, F2, F4, F6, F8, FT7, FC5, FC3, FC1, FC2, FC4, FC6, FT8, T7, C5, C3, C1, C2, C4, C6, T8, TP7, CP5, CP3, CP1, CP2, CP4, CP6, TP8, P7, P5, P3, P1, P2, P4, P6, P8, PO7, PO3, PO4, PO8, O1, O2), and the right mastoid A2. During measurement all electrodes were referenced to the left mastoid (A1) and one of the electrodes in the cap (AFz) was used as ground. Offline, EEG data were re-referenced to the average of the right and left mastoids. Blinks, vertical and horizontal eye-movements were measured by bipolar electrodes placed above and below the left eye and at the outer canthi of both eyes. All electrode impedances were kept below 10 kΩ, with the exception of the reference and ground electrodes, which were held below 5 kΩ. Signal acquisition was accomplished using Brainamp amplifiers and Brain Vision Recorder software (version 1.10). EEG and EOG signals were continuously sampled at 500 Hz with a high-pass filter of 0.05 Hz and a low-pass filter of 250 Hz.

ERP analysis was done in Neuroscan 4.3.1. The continuous EEG was divided into 480 epochs of 1250 ms, from 200 ms prestimulus to 1050 ms poststimulus, all aligned to a baseline from −200 to 0 ms preceding the memory array, and low-pass filtered offline at 30 Hz. First, vertical (blinks) and horizontal electro-oculogram (VEOG and HEOG) artifacts were removed from the data by applying an eye-movement correction algorithm [46]. For the computation of regression coefficients between VEOG and the EEG-signals at the different electrodes, adequate eye blinks were manually selected and transmission coefficients were computed on the basis of these selected trials. In a similar way, by manually selecting horizontal eye movements separately for right- and left cued displays for each individual, regression coefficients between HEOG and the EEG-signals at the different electrodes were computed. All transmission coefficients were carefully checked to have strengths, signs and topographies congruent with expected patterns for vertical and horizontal movements before they were applied to remove eye blinks and horizontal eye movements from the EEG through the Semlitsch et al. [46] procedure. After EOG-artifact removal, epochs still containing artifacts exceeding ±75 µV were rejected from the database. Whereas the above procedure should have removed all HEOG activity from the EEG signal, to be sure that our CDA load and distracter effects were not due to any residual HEOG activity we performed an extra check by calculating the correlations between CDA and HEOG in each condition between 450 and 825 ms. No significant correlations between CDA and HEOG in T1D0, T1D2 or T3D0 were found in the four groups. Next, average ERPs were computed separately for each subject in three different task conditions: (1) one target square only (T1D0; where T is the number of targets and D is the number of distracters), (2) one target square plus two distracter rectangles (T1D2), (3) three target squares only (T3D0). The fourth condition (T3D2) was not included in the analyses since its number of shapes (five) exceeds the maximum memory capacity of four items. In the averaging procedure, only trials with correct responses were included. There was a maximum number of 120 trials in each task condition. The minimum number of included trials in each condition was 20. Across conditions, the mean number of artifact-free EEG epochs, after exclusion of incorrect trials, contained in the single-subject averages was 78 trials (SD = 23) in the control adolescent group, 78 trials (SD = 15) in the ADHD adolescent group, 101 trials (SD = 17) in the control adult group, and 100 trials (SD = 12) in the ADHD adult group. Note that in the adolescent groups most trials were lost to higher numbers of trials with incorrect responses and not to lower quality data.

We computed contralateral waveforms by averaging the activity recorded at right hemisphere electrode sites when subjects were cued to remember the left side of the memory array with the activity recorded from the left hemisphere electrode sites when they were cued to remember the right side. CDA was measured at 7 posterior parietal and lateral occipital electrode sites (P1/2, P3/4, P5/6, P7/8, PO3/4, PO7/8, O1/2) based on visual inspection of where largest CDA effects were present in our data (and on distributions reported in an earlier developmental CDA paper [47]). CDA was computed as the difference in mean amplitude between the ipsilateral and contralateral waveforms; this is exactly similar to the CDA calculation methods used in [26], [28]. Since the differences between conditions generally seem to decrease over time in the delay period (see [48] for similar results) and different effects across the retention period were clearly visible in the CDA for the different groups (comparable to some other CDA studies including groups of different ages, see [49], two measurement windows were chosen for analyses, an early window of 450–550 ms and later window of 550–825 ms after the onset of the memory array.

### Statistical Analysis

#### Behavioral measures

Effects of Age (adolescents vs. adults) and Diagnosis (control vs. ADHD) and possible interactions between both on verbal and VSWM-capacity were tested by performing 2×2 univariate ANOVA analyses separately for forward, backward and standardized (including both forward and backward scores; for computation see WISC-III/WAIS-III manual) digit span tests (verbal WM), and *K* in T1D0, T1D2 and T3D0 conditions (VSWM).

To test for group differences in filtering efficiency in the change detection task planned ANOVA analyses with factors Trial-type (either T1D0 vs. T1D2 or T1D2 vs. T3D0), Age (adolescents, adults) and Diagnosis (control, ADHD) were performed on reaction time (RT) and accuracy (% correct). With worse filtering efficiency T1D0–T1D2 RT differences will increase and T1D2–T3D0 RT differences will decrease due to longer search times in T1D2 (due to unnecessary storage of distracters). In the same way T1D0–T1D2 accuracy differences will increase and T1D2–T3D0 accuracy differences will decrease in case of inefficient filtering. Differences in unnecessary storage (*K*-T1D0–*K*-T1D2) between Age and Diagnosis groups were tested with a 2 (adolescents vs. adults)×2 (control vs. ADHD) univariate ANOVA.

#### ERP measures

For the parietal/occipital CDA windows, mean amplitudes were compared across conditions by repeated measures ANOVAs. Three planned mixed model ANOVA’s were performed in both time windows, with a within-subject factor Trial-type with two levels (T1D0 & T1D3 or T1D0 & T1D2 or T1D2 & T3D0) and two between-subjects factors Age (adolescents, adults) and Diagnosis (control, ADHD). In case of significant Age×Diagnosis×Trial-type interactions or Age×Diagnosis interactions, post-hoc tests were performed for the separate Age groups. In case of Age×Trial-type or Diagnosis×Trial-type interactions, further testing was done to reveal possible effects of Trial-type.

#### Correlations

Correlations between behavioral measures of storage capacity, filtering efficiency (T1D0–T1D2 RT and accuracy differences or unnecessary storage) and CDA amplitude differences in T1D0 and T1D2 for the early and late CDA window were calculated using Pearson correlation coefficients.

## Results

### Behavioral Results

#### Developmental differences in verbal (digit span) and visuospatial span (Cowan’s *K*) in ADHD and controls

**Figure 2:**
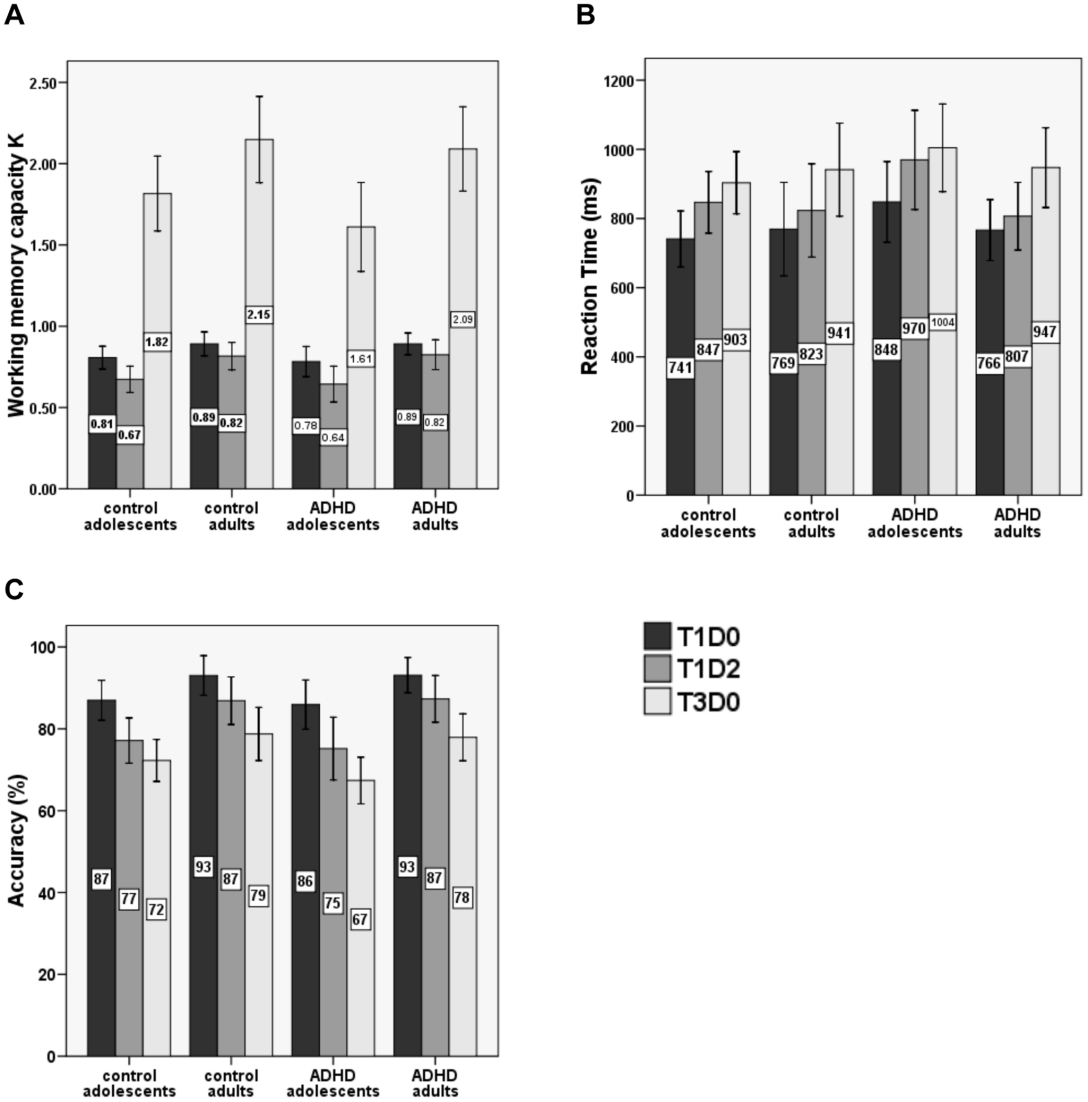
Behavioral data from the VSWM change detection task. Bar graphs of (A) Cowan’s *K*, (B) average reaction times (in ms), and (C) percentage of correct responses for control and ADHD adolescents and adults in T1D0 (one target), T1D2 (one target, two distracters) and T3D0 (three targets) conditions of the VSWM change detection task. Error bars indicate 95% confidence intervals.

Forward, backward and standardized digit spans were collected for all groups to obtain measures of verbal WM span, and visuospatial WM span was measured by computing *K* in T1D0, T1D2 and T3D0 conditions of the VSWM task. For means and standard deviations (SD’s) of scores in the digit span task see Table 1. For mean *K* scores (and SD’s) in the VSWM task see Table 2 and Fig. 2A.

**Table 2:** Group means (standard deviations between brackets) of WM-capacity *K* in T1D0, T1D2 and T3D0 conditions of the VSWM change detection task for adolescents and adults in the control and ADHD groups.

	WM-capacity *K* (visuospatial)		
	T1D0	T1D2	T3D0
**Control Adolescents**	.81 (.15)*	.67 (.17)**	1.82 (.48)**
	Range:.42–.98	Range:.33–.93	Range:.78–2.50
**ADHD** **Adolescents**	.78 (.17)*	.64 (.20)**	1.61 (.49)**
	Range:.49–.97	Range:.31–.93	Range:.73–2.30
			
**Control Adults**	.89 (.14)*	.82 (.16)**	2.15 (.50)**
	Range:.43–.99	Range:.39–.98	Range: 1.20–2.83
**ADHD Adults**	.89 (.13)*	.82 (.18)**	2.09 (.50)**
	Range:.51–1.0	Range:.22–.98	Range:.95–2.80

NB: stars indicate significant Age differences (*P* values):

* *P<*.01;

** *P*<.001.

The adolescent groups had significantly lower verbal span than the adult groups as shown by forward, backward and standardized digit span (main effects of Age: forward digit span: F(1,63) = 10.4, *P*<.005; backward digit span: F(1,63) = 20.1, *P*<.0001; standardized digit span: F(1,63) = 12.7, *P*<.001). The ADHD groups only showed lower forward span compared to the control groups, irrespective of age (main effect Diagnosis: F(1, 63) = 4.5, *P*<.05).

Adolescents had significantly lower VSWM-capacity, reflected by lower *K* scores than adults in all three conditions (main effects of Age: T1D0 (F(1,63) = 7.4, *P*<.01), T1D2 (F(1,63) = 14.2, *P*<.001) and T3D0 (F(1,63) = 11.3, *P*<.005). No differences in *K-*scores between ADHD and control groups (no Diagnosis or Age×Diagnosis effects) were found.

#### Effect of distracters on the speed (RT) and accuracy of change detection performance

For both RT and accuracy data two planned 2 (Age; adolescents, adults)×2 (Diagnosis; ADHD, control)×2 (Trial-type; T1D0–T1D2 OR T1D2–T3D0) ANOVA’s were carried out to test for effects of distracters on VSWM performance (see Fig. 2B and 2C for means of RT and accuracy 95% confidence intervals respectively).

For reaction times, the first comparison (T1D0 vs. T1D2) yielded a significant Age×Trial-type interaction effect (F(1,63) = 7.9, *P*<.01), indicating that adolescents showed stronger RT increases from T1D0 to T1D2 (due to the presence of distracters) than adults. The T1D2 vs. T3D0 comparison also yielded a significant Age×Trial-type interaction (F(1,63) = 11.5, *P*<.005), showing that reaction time increased less from T1D2 to T3D0 in adolescents than in adults (see Fig. 2, panel B). No main effects or interactions involving the Diagnosis factor were found for reaction time.

For accuracy, the first comparison (T1D0 vs. T1D2) yielded a significant Age×Trial-type interaction effect (F(1,63) = 7.1, *P*<.01), showing a steeper decline in memory accuracy from T1D0 to T1D2 (with distracters) in adolescents than adults (see Figure 2C). The second comparison (T1D2 vs. T3D0) yielded a main effect of Trial-type (F(1,63) = 56.4, *P*<.00001) indicating that averaged over all groups, subjects’ performance was less accurate in the T3D0 condition than in the T1D2 condition. Whereas the size of this decline in accuracy from T1D2 to T3D0 was not significantly different between adolescents and adults (no Trial-type×Age interaction), a main effect of Age (F(1,63) = 13.8, *P*<.001) did show that adolescents generally made more errors than adults in both the distracter (T1D2) and the high WM-load (T3D0) condition. There were no effects of Diagnosis.

As indicated earlier, worse filtering efficiency will become visible as larger T1D0–T1D2 performance (or CDA) differences and smaller T1D2–T3D0 performance (or CDA) differences; which is exactly the pattern that was found for the RT results (and partly also the accuracy results) in adolescents (when compared to adults).

Finally, a main Age effect (F(1,63) = 6.9 *P*<.05) for the unnecessary storage measure showed significantly higher storage of distracters in adolescents (.14) than in adults (.07). There were no differences in unnecessary storage between ADHD and control groups (no effect of Diagnosis or Diagnosis×Age interaction was found).

### CDA Results

**Figure 3:**
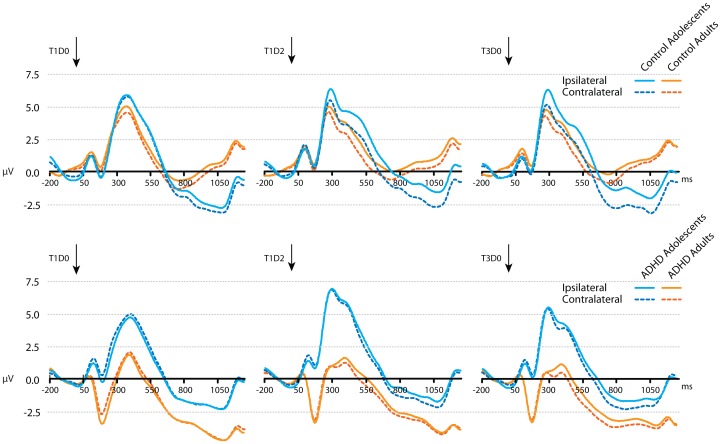
Average ERP activity during the VSWM change detection task. Ipsilateral (solid lines) and contralateral (dashed lines) activity, averaged across occipital and posterior parietal electrode sites (P1/2, P3/4, P5/6, P7/8, PO3/4, PO7/8, O1/2) for control and ADHD adolescents and adults, in conditions T1D0, T1D2 and T3D0. Arrows indicate presentation of the memory array.

**Figure 4:**
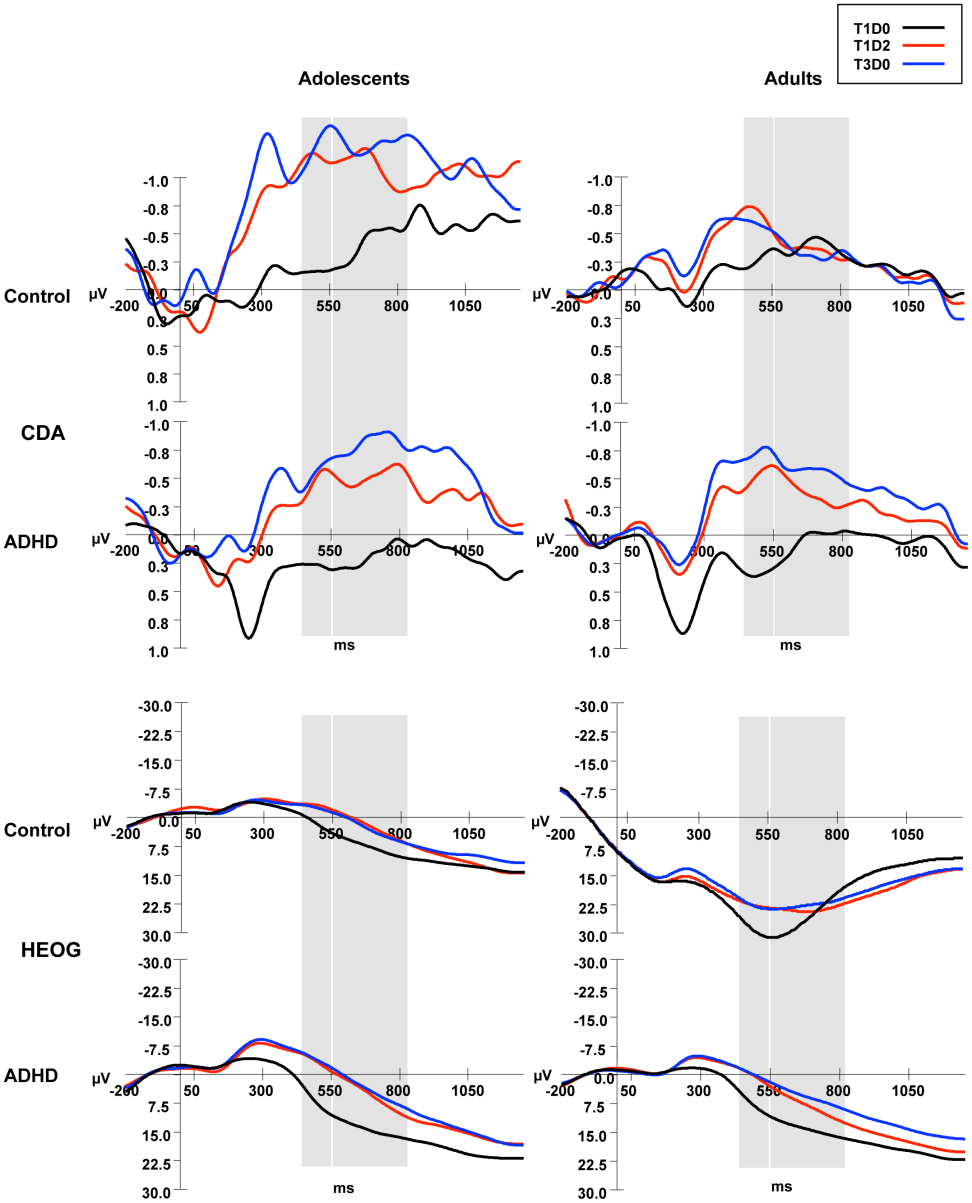
Average CDA and HEOG activity during the VSWM change detection task. CDA activity (computed by subtracting ipisilateral from contralateral activity) and HEOG activity ((HEOG left visual field trials*−1+ HEOG right visual field trials)/2) were smoothed with a 6 Hz low-pass filter, time-locked to the memory array and averaged across occipital and posterior parietal electrode sites for control and ADHD adolescents and adults, in conditions T1D0, T1D2 and T3D0. Grey bars indicate time windows for analysis: 450–550 ms and 550–825 ms.

Contralateral and ipsilateral activity averaged for occipital and parietal sites (P1/2, P3/4, P5/6, P7/8, PO3/4, PO7/8, O1/2) in the adolescent and adult groups for the three task conditions are shown in Fig. 3. Grand ERP averages of CDA (contralateral minus ipsilateral activity) at these occipital and parietal sites in the four groups for the three conditions are depicted in Fig. 4. Mean CDA amplitudes and SD’s in the predefined 450–550 and 550–825 ms time windows per task condition and Age groups are shown in Table 3.

**Table 3:** CDA data from the VSWM change detection task.

		T1D0	T1D2	T3D0
**CDA (450–550** **ms)**	**Control Adolescents**	−0.3 (1.41)	−1.21 (1.25)	−1.24 (1.44)
	**ADHD Adolescents**	0.23 (0.95)	−0.47 (1.31)	−0.54 (1.03)
	**Control Adults**	−0.28 (0.56)	−0.67 (0.62)	−0.60 (0.56)
	**ADHD Adults**	0.35 (0.35)	−0.54 (0.86)	−0.76 (0.81)
**CDA (550–825** **ms)**	**Control Adolescents**	−0.39 (1.43)	−1.11 (1.10)	−1.31 (1.39)
	**ADHD Adolescents**	0.14 (1.07)	−0.51 (1.11)	−0.82 (0.99)
	**Control Adults**	−0.38 (0.53)	−0.35 (0.40)	−0.38 (0.51)
	**ADHD Adults**	0.06 (0.44)	−0.37 (0.68)	−0.59 (0.67)

Mean CDA amplitude (standard deviations between brackets) at posterior parietal and lateral occipital electrode sites (µV) over the indicated time windows, in the three conditions for adolescents and adults in the control and ADHD groups.

#### Effects of load and distracters on CDA in the 450–550 ms maintenance interval

To test for *group differences in effects of increasing working memory load* (maintenance of one vs. three items), mean amplitudes of parietal-occipital CDA in the first time window (450–550 ms) in T1D0 and T3D0 conditions were entered into a 2 (Age; adolescents vs. adults)×2 (Diagnosis; ADHD vs. control)×2 (Trial-type) repeated measures analysis. A significant interaction between Age, Diagnosis and Trial-type was found (F(1,63) = 5.1, *P*<.05); this interaction was followed up by testing for Trial-type×Diagnosis effects in adolescent and adult groups separately. In the adolescent groups there was a main effect of Trial-type (F(1,32) = 18.5, *P*<.001) signifying a CDA amplitude increase with increasing load, but there was no interaction with Diagnosis among adolescents. In the adult groups however, there was a significant Trial-type×Diagnosis interaction (F(1,31) = 12.7, *P*<.01), showing a larger load-related CDA increase in adults with ADHD (Trial-type effect: F(1,16) = 38.4, *P*<.0001) than in typically developed adults (F(1,15) = 6.6, *P*<.05). No other main or interaction effects were found. To test whether the CDA increase with load was also larger in adolescents than control adults, two extra comparisons were done (control adolescents vs. control adults and ADHD adolescents vs. control adults). The effect of Trial-type on CDA was different for control adolescents and control adults (interaction Trial-type×Age: F (1,33) = 4.4, *P*<.05). There was however no such difference between ADHD adolescents and control adults, but only an overall increase in CDA amplitude in these groups for increasing load (Trial-type-effect: F (1,29) = 13.7, *P*<.001).

To test for *group differences in effects of distracters* on CDA amplitude, similar 2×2×2 repeated measures analyses of variance were conducted for T1D0 vs. T1D2 and for T1D2 vs. T3D0 conditions. The comparison between T1D0 and T1D2 only showed a main effect of Trial-type (F(1,63) = 40.6, *P*<.0001); T1D2 CDA amplitude was higher than T1D0 CDA amplitude (this effect was also found in the separate groups at *P*<.05 level, although at *P* = .057 significance level in ADHD adolescents). Furthermore, a main effect of Diagnosis (F(1,63) = 4.8, *P*<.05) indicated larger CDA amplitudes in the control groups than in the ADHD groups. No other significant main or interaction effects were found, except for a marginally significant interaction Age×Diagnosis×Trial-type (F(1,63) = 3.3, *P* = .075) which was not further tested. For the T1D2–T3D0 comparison, no main or interaction effects for Trial-type, Age or Diagnosis were found.

The pattern of T1D0< T1D2 = T3D0 CDA amplitude that was found in all groups (although marginally significant for the T1D0–T1D2 comparison in ADHD adolescents) in this early encoding window shows that there was distracter encoding (suboptimal filtering).

#### Effects of load and distracters on CDA during the 550–825 ms maintenance interval

The test for *group differences in effects of increasing load* (T1D0 vs. T3D0) showed a main Trial-type effect (F(1,63) = 24.1, *P*<.0001) qualified by an Age×Trial-type interaction (F(1,63) = 5.8, *P*<.05), showing that adolescents had a larger increase in CDA amplitude when three items had to be remembered compared to one than adults. No main effect or interaction effects involving the factor Diagnosis were found).

The test for *group differences in effects of distracters* on CDA amplitude in this later processing window yielded two significant effects for the T1D0–T1D2 comparison. First, a main marginally significant effect of Diagnosis (F(1,63) = 3.9; *P* = .053) indicated that adolescents and adults with ADHD had overall smaller T1D0 and T1D2 CDA amplitudes than typically developing adolescents and adults, just as in the earlier encoding window. Second, a significant Age×Trial-type interaction (F(1,63) = 4.5; *P*<.05) indicated that in this processing window different effects of distracters were found on the CDA in younger and older participants. Follow-up tests resulted in a main Trial-type effect in adolescents (F(1,32) = 11.4; *P*<.005), because of significantly higher parietal-occipital CDA amplitudes in T1D2 than in T1D0 indicative of distracter maintenance (e.g. suboptimal filtering). In adults with and without ADHD however, this CDA amplitude enhancement was only marginally significant (Trial-type effect: F(1,31) = 3.7; *P* = .064). For the T1D2–T3D0 comparison main effects of Trial-type and Age were found. The Trial-type effect (F(1,63) = 4.3, *P*<.05) indicated that, averaged over all groups, the T3D0–CDA was significantly higher than the T1D2–CDA, which is indicative for some filtering of distracters during maintenance. Second, a main effect of Age (F(1,63) = 6.0, *P*<.05), indicated that CDA amplitudes (in both T1D2 and T3D0 conditions) were larger in adolescents than in adults, irrespective of Diagnosis.

Thus, in this later maintenance interval, in adolescents a T1D0< T1D2 CDA pattern was found, indicating that they maintained distracters in WM during the delay, since T1D0 and T1D2 CDA’s did not overlap. Adults, however, showed a T1D0 = T1D2 pattern that has in earlier studies been associated with efficient distracter filtering (see 28).

### Correlations between Performance and Electrophysiological CDA Measures

**Figure 5:**
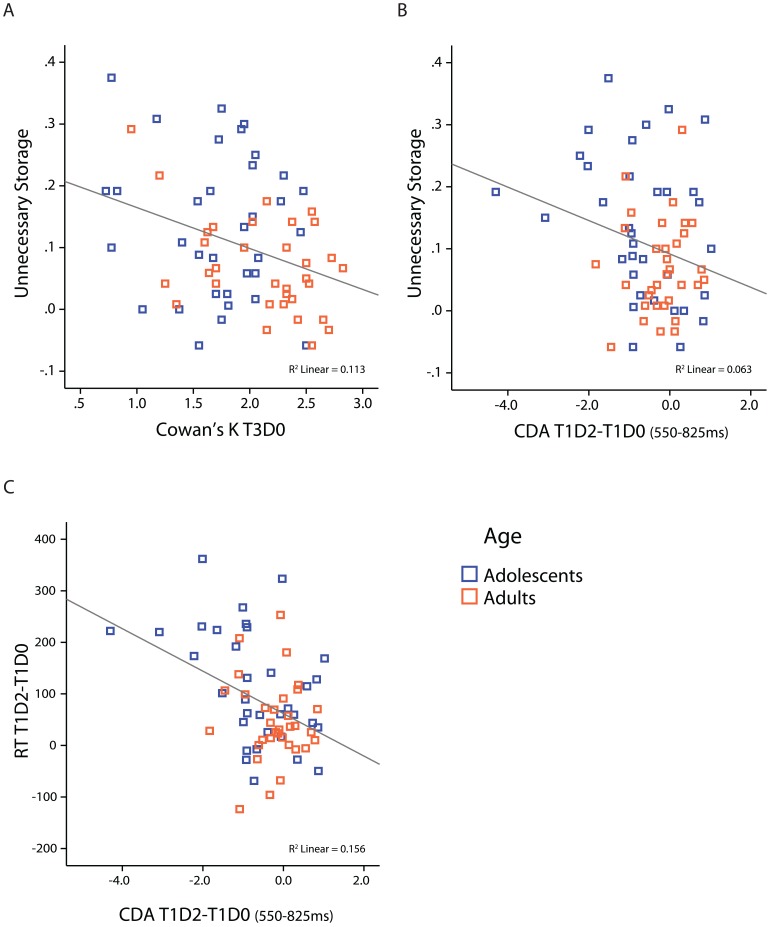
Scatterplots of significant correlations between behavioral and CDA measures. (A) Correlation between K_T3D0 and unnecessary storage (K- T1D0 minus K-T1D2) for all subjects. (B &C) Correlation between distracter-related parietal/occipital CDA effects (CDA_T1D2 minus CDA_T1D0) from 550–825 ms (x–axis) and unnecessary storage (K_T1D0– K_T1D2; panel B) in control and ADHD groups, or RT distractereffects (RT_T1D2 minus RT_T1D0; panel C) and unnecessary storage (K_T1D0– K_T1D2; panel E) in control and ADHD groups. Fit lines are for the whole group, colors indicate adolescents and adult groups.

To replicate relations between performance and CDA measures of WM-capacity and filtering efficiency reported in earlier work, correlation analyses were performed across all subjects to take into account all individual variance in WM-capacity. Fig. 5 depicts the most important significant correlations across all subjects.

Including all adults and adolescents, individuals with higher VSWM span also had higher verbal working memory span (but not short-term memory span); *K* scores in the highest load condition (K_T3D0) were correlated with backward (r(65) = .409, *P = *.001), and standardized (r(65) = .297, *P = *.015) digit span scores, but not with forward digit span scores (*P = *.117).

Performance (*K*) measures of storage capacity in the VSWM task were also related to performance measures of filtering efficiency in the same task. A significant negative correlation of r(65) = -.336, *P = *.005 was found between *K* (T3D0) and the unnecessary storage measure (*K*_T1D0 minus *K*_T1D2) when all subjects were included, indicating that individuals with higher VSWM-capacity stored less irrelevant items in memory (see Fig. 5A). Because the ANOVA analyses showed Age differences in *K_*T3D0 and Unnecessary Storage, correlations were also computed within adolescent and adult groups; the correlation was only significant in adults (r(31) = -.446, *P = *.009). The absence of a significant correlation in adolescents is probably due to lower variance in *K*-scores in adolescents (e.g. less scores in the upper range) due to immaturity in WM capacity (see Fig. 5A).

Finally, correlations were found between performance and CDA measures of filtering efficiency. Firstly, the unnecessary storage measure showed a negative correlation with distracter-related CDA effects (T1D2 amplitude minus T1D0 amplitude) across all subjects in the late part of the maintenance window from 550–825 ms (r(65) = -.250, *P = *.041) (see Fig. 5B). Secondly, a similar negative correlation was found between distracter-related RT increases (RT_T1D2 minus RT_T1D0) and distracter-related CDA increases across all subjects in the late maintenance window (550–825 ms) (r(65) = -.395, *P = *.001) (see Fig. 5C). When tested within adolescent and adult groups, after correction for multiple testing, only the correlation between distracter-related effects on RT and CDA in the late (550–825) maintenance window (r(32) = -.457, *P = *.007) in adolescents remained significant. This fits the developmental findings from the ANOVA analyses that only adolescents maintained distracters in memory (had higher T1D2 than T1D0 CDA amplitude) during the late phase of the maintenance interval with negative consequences for performance. The absence of a correlation in the late window in adults is most likely the result of low variance in T1D2–T1D0 difference scores (waves were overlapping in adults; see ANOVA analyses described above) providing evidence for some distracter filtering; there was a trend towards a significant correlation in adults in the early window (*P = *.09) that did however not survive correction for multiple testing.

## Discussion

The present study investigated the development of visuospatial working memory (VSWM) capacity and its relations with filtering efficiency during adolescence in ADHD. Memory impairments in WM tasks and filtering deficits in selective attention tasks have independently been reported in ADHD but their relation has never been directly investigated. To fill this gap, in the present study performance and CDA activity were measured in a VSWM change detection task with targets and distracters in adolescents and adults with and without ADHD that were matched on educational background and IQ.

### Filtering Efficiency in a VSWM Task in Adolescents and Adults with ADHD

The WM performance of all subjects suffered from the presence of distracters in the memory set as shown by the unnecessary storage and reaction time measures. However, this distracter interference was larger in adolescents than adults. These developmental effects did not differ between non-ADHD and ADHD groups. The CDA-ERPs were calculated separately for the distracter-present and distracter-absent trials in early and late intervals of the delay period to get insight into online filtering and storage of distracters in the brain. In an early phase of the retention interval during which stimuli were encoded/maintained in WM (450–550 ms after presentation of the memory display), CDA amplitude was increased when distracters were present (vs. absent) in all groups. The CDA response to the distracter display (T1D2; containing one target and two distracters) was significantly larger than the CDA response to the display with only one target without distracters (T1D0), and overlapped with the CDA in response to the three target-no distracters (T3D0) display, suggesting that all subjects initially encoded the distracters, irrespective of Diagnosis or Age. This pattern changed during a later part of the delay interval (550–825 ms). An interaction between Age and Trial-type for this late CDA showed that adolescents maintained the distracters in memory during the entire delay interval (T1D0–T1D2 CDA differences sustained) whereas in adults the distracter-related T1D2-CDA dropped in amplitude to the level of T1D0. This finding provides evidence for some distracter filtering in adults (with and without ADHD) during the later part of the delay interval. In the original studies including university students with relatively high WM-capacity (e.g. high *K-*scores) CDA results were indicative of filtering already occurring early in the retention interval [28]. Later studies including populations with lower *K*-scores and different ages have however also reported CDA results indicative of absent filtering or filtering during a later phase of the retention interval, just as in our study [49]. As opposed to in the earlier interval, in the later part of the retention window there also was a significant main effect of Trial-type indicating that T3D0-CDA was larger than T1D2-CDA, a pattern indicative of some filtering (see [28]). This Trial-type effect was however not very strong and was not significant when tested in the separate groups.

This combination of behavioral and CDA findings leads to the conclusion that adolescents’ WM performance suffers more from the presence of distracting information than that of adults due to immature filtering mechanisms, as was shown by the CDA that is assumed to reflect the online storage of targets/distracters in memory. Correlation analyses confirmed this, by showing that during the late part of the maintenance interval, in which significant developmental differences in the ability to filter out distracters were found, behavioral and CDA measures of filtering efficiency were significantly related. Subjects with the largest distracter-related response in the CDA also had showed the largest distracter interference on behavior (e.g. larger unnecessary storage score and largest increases in memory decision speed). We suggest that still immature functioning of dorsolateral prefrontal cortex (DLPFC) or its connections with posterior cortex might underlie immature filtering in adolescents in WM tasks [50], [51] because of recent evidence that activation in this region is related to executive controlled distracter suppression during spatial memory encoding/maintenance [52]. There is evidence that low working memory functioning in adolescence might be related to impulsive behavior through reduced action control exerted by the DLPFC [53], [54]. This was based on the finding of negative relations between working memory capacity and reports of “acting without thinking” behaviour in adolescents, the latter in turn being linked to higher levels of risk behaviour and externalizing problems [53], [54]. In light of this, it seems important to monitor for lower than normal working memory functioning (both in capacity and filtering) before adolescence in order to remediate such WM-deficiencies early on (by for example working memory training, see [55]) to prevent possible adverse behavioural outcome during later adolescence.

Performance and CDA measures did not provide evidence for a developmental lag in filtering efficiency in a VSWM task in adolescents or adults with ADHD when compared to education/IQ- and age-matched controls. Whereas deficits in early attentional filtering have been reported in children with ADHD in previous ERP studies using classical auditory or visual two-channel filtering tasks [32], [34] or a visual selective memory search task [56], selective attention ERP studies in adolescents or adults with ADHD are lacking. In one behavioural study [57], early stage attentional filtering in adolescents with ADHD was studied using an attentional blink task. Supporting our results, no evidence for attentional filtering deficits in adolescents with a DSM-diagnosis of the inattentive or combined ADHD subtype were found, the same subtypes as included in our study. There are large differences however in the type of attention tasks used among these studies and also groups were not always matched on education level/IQ. The present study shows that the development of filtering efficiency in a VSWM task in adolescents and adults with ADHD is not different from that in healthy adolescents and adults when subjects are carefully matched on IQ and education level.

The only significant effect of Diagnosis was the overall smaller CDA amplitude when a low load of only one item had to be stored in visual working memory in adolescents and adults with ADHD than in age-matched controls. Because the CDA is the result of the subtraction of ipsilateral from contralateral activity, these smaller CDA amplitudes could have been the result of lower contralateral or larger ipsilateral activity in response to the bilaterally presented stimulus set. Wandering of attention to the non-cued side of the display (e.g. that side one should not attend) would cause relatively enhanced ipsilateral activity, reducing lateralized CDA activity. The effect of irrelevant information in the non-cued hemifield on the ipsilateral CDA in healthy adults was recently investigated by varying the number of items on the non-cued side of the screen [58]. It was found that ipsilateral CDA activity was increased by the number of irrelevant items presented in the non-cued hemifield, but only when the to-be-stored WM load in the cued-display was low (1 item). The authors explained this by assuming that capacity that was not used for the processing of information in the contralateral hemifield “spilled over” to the non-cued side, despite instruction to keep focused on the cued hemifield. Post-hoc analyses of our data confirmed that in the early encoding window, ipsilateral activity to the 1-target (T1D0) display was increased in the ADHD groups compared to in the control groups (effect Diagnosis *p* = .052), whereas this was not the case for the T1D2 display (*p* = 0.23). This is consistent with the view that ADHD patients had more problems than controls with focusing attention on the cued-hemifield when WM encoding/processing demands were relatively low. Higher distractibility particularly with *low* perceptual load was earlier demonstrated in children with ADHD [59] and in healthy adult subjects with high (vs. low) distractibility scores on a Cognitive Failures Questionnaire [60].

Although purely speculative at this stage, this higher level of wandering of attention away from the to-be-attended side of the stimulus display when the to-be maintained memory load was low might be due to an increased tendency for mind wandering in our ADHD patients. Mind wandering has been shown to be a fairly stable cognitive characteristic, its frequency predicting difficulties with executive control in daily life and in laboratory tasks [61]. Fitting our results, there is also evidence that mind wandering especially occurs in relatively easy tasks since in this case task demands and task-unrelated thoughts do not compete for resources, increasing the room for the mind to wander [62]. The biological basis for such increased mind wandering might lie in insufficient prefrontal control over activity of the default mode network in the brain of adolescents and adults with ADHD [63]–[65], since higher activity in this network has been associated with higher levels of mind wandering [66], [67].

### VSWM-capacity in Adolescents and Adults with ADHD

Besides studying developmental differences in WM- distracter filtering between ADHD and non-ADHD groups, the present study also investigated potential differences in VSWM capacity. Prior studies that provided evidence for lower VSWM span in ADHD especially during late childhood/adolescence, included subjects that were not matched on IQ [9], [20]. The present data (Cowan’s *K* or CDA) provide no evidence for developmental differences in visuospatial span or storage space between adolescents and adults with and without ADHD when matched on IQ. A main effect of Age however indicated that independent of diagnosis, adolescents had lower *K*-scores than adults, pointing to similar developmental immaturity of VSWM span in adolescent ADHD and non-ADHD groups. Some other studies using change detection tasks have reported mature VSWM-capacity around age 10–12 [68], [69]. The late development of VSWM span in the present study might be due to the relatively low IQ levels of our subjects since IQ and WM-performance are closely related [21], [22]. Scores on the digit span test, a standardized measure of verbal WM-span, indeed indicated that all our adolescents (with a mean age of 14.8 years) scored below their norm groups, irrespective of diagnosis. That is, the typically developing adolescents had a mean digit span score of 13.7, which is at the level of 13-year-olds’ span and the adolescents with ADHD had a mean score of 12.8, which is at the level of 11.5-year-old typically developing children. The present data show that matching on IQ is highly important when investigating developmental differences in WM performance between non-clinical and clinical groups.

CDA amplitudes in the early part of the retention interval (450–550 ms) displayed increased activity when more memory items were present in the memory display (one vs. three) in all groups. This load-related CDA increase was higher in adults with ADHD than in control adults during the early encoding window, as indicated by a three-way interaction between Trial-type, Diagnosis and Age. During the later maintenance window the load-related CDA increase was larger in adolescents than adults (irrespective of diagnosis), as shown by an interaction between Age and Trial-type. Such higher load-related CDA responses in adults with ADHD and in adolescents are not likely to be related to the storage of a higher number of items in WM, since the CDA-increase is not accompanied by better performance. Multiple recent change detection studies however point to an important role of (spatial) attention in the modulation of load-related CDA responses. In an fMRI study [70], attention to items in the memory set was manipulated and it appeared that an active neural trace was only elicited by items within the focus of attention. But most importantly, their data suggested that for accurate memory of memory loads that do not exceed one’s capacity, allocation of (spatial) attention for maintenance (accompanied by neural activation) is not always necessary. These findings suggest that there might be relatively smaller CDA responses with higher loads in subjects with higher capacity (due to lower needs for allocation of attention) and such an explanation would fit our findings of lower K accompanied by higher load-related CDA increases in our adolescents. Thus possibly, the sustained CDA response with load in adolescents indicates that adolescents needed more spatial attention (possibly for spatial rehearsal) than adults to refresh the (location of) the items in memory. More support for a relation of CDA amplitude with spatial attention is given by several recent studies (e.g. [71], [72]. Interestingly, similar larger load-related CDA responses have recently been reported in children compared to adults in another developmental change detection study [47], although these differences were only found when stimulus presentation times were relatively long (e.g. 500 ms). With short presentation times of only 100 ms, comparable to those used in the present study, load-related CDA effects were no longer present in these younger children, because according to the authors, children might have “given up” in high load conditions due to time constraints. The fact that in the present study load effects on the CDA were present in adolescents even with 150 ms display presentation times suggests that important development of top-down control or the ability to allocate attention resources for WM maintenance takes place between childhood and adolescence.

Last, across all subjects, the measure of cost of distracters in accuracy and the unnecessary storage *K-*score was negatively correlated with the *K-* capacity score. This shows that individuals with higher WM-capacity experienced less interference from distracters than lower capacity participants. This relation between WM-capacity and filtering efficiency was previously also found [73] in adults, and emphasizes the interaction between storage capacity and efficient filtering. Both typically developing adolescents and adolescents with ADHD show impaired performance on *both* measures compared to adults with or without ADHD in the present study.

### Conclusion

Behavioral and CDA results from the present study indicated that filtering efficiency in a VSWM task was still immature in adolescents and this developmental pattern was similar for subjects with and without an ADHD diagnosis of the combined or inattentive type. Electrophysiological brain (CDA) activity suggested that only adults were able to block irrelevant information from storage in visual memory (thereby improving performance), but only during a later stage of the maintenance interval. Also, evidence for ongoing development of VSWM capacity during adolescence was found, as indicated by the behavioural and electrophysiological results associated with maintenance of different WM loads. Again, VSWM-capacity levels in typically developing adolescents and adults did not differ from that of age matched-peers with an ADHD diagnosis. Together, these findings suggest that there is no developmental lag in visuo-spatial WM-filtering or capacity in adolescents or adults with ADHD. Only when the WM load was low (1 item), adolescents *and* adults with ADHD showed increased brain activity ipsilateral to the cued hemifield, possibly reflecting an inability to ignore irrelevant information in the non-cued hemifield when processing demands are relatively low. This did however not result in worse performance.
